# Fabrication of a Food Nano-Platform Sensor for Determination of Vanillin in Food Samples

**DOI:** 10.3390/s18092817

**Published:** 2018-08-27

**Authors:** Vinod Kumar Gupta, Hassan Karimi-Maleh, Shilpi Agarwal, Fatemeh Karimi, Majede Bijad, Mohammad Farsi, Seyed-Ahmad Shahidi

**Affiliations:** 1 Department of Applied Chemistry, University of Johannesburg, Johannesburg 17011, South Africa; shilpi.agarwal2307@gmail.com; 2 Laboratory of Nanotechnology, Department of Chemical Engineering, Quchan University of Technology, Quchan 94771-67335, Iran; fkm024@gmail.com; 3 Department of Agriculture, Sari Branch, Islamic Azad University, Sari 48161-19318, Mazandaran, Iran; majedebijad@yahoo.com (M.B.); mfarsi2008@gmail.com (M.F.); 4 Department of Food Science and Technology, Ayatollah Amoli Branch, Islamic Azad University, Amol 46311-39631, Mazandaran, Iran; a.shahidi@iauamol.ac.ir

**Keywords:** vanillin, NiO-SWCNTs nanocomposites, 1-butylpyridinium hexafluorophosphate

## Abstract

Herein, we describe the fabrication of NiO decorated single wall carbon nanotubes (NiO-SWCNTs) nanocomposites using the precipitation method. The synthesized NiO-SWCNTs nanocomposites were characterized by X-ray diffraction (XRD) and Transmission electron microscopy (TEM). Remarkably, NiO-SWCNTs and 1-butylpyridinium hexafluorophosphate modified carbon paste electrode (CPE/NiO-SWCNTs/BPrPF6) were employed for the electrochemical detection of vanillin. The vanillin sensor showed an ultra-high sensitivity of 0.3594 μA/μM and a low detection limit of 0.007 μM. In the final step, the NiO-SWCNTs/BPrPF6 was used as the suitable tool for food analysis.

## 1. Introduction

The food analysis is an important strategy for the investigation of food quality [1]. The forbidden additives must be checked by an analytical sensor before consuming by customer [2]. Ensuring the safety of food can be checked by the analysis of food compounds. Although numerous analytical methods are available to analyze foods—including gas chromatography [3], capillary electrophoresis [4], spectrophotometry [5], resonance Raman spectroscopy [6], high-performance liquid chromatography [7], and electrochemical sensors [8,9,10,11,12,13]. However, electrochemical sensors are better suited for this goal due to portable ability, fast response, easy operation, and low cost [14,15,16,17,18,19,20]. Recently, chemically modified sensors improved on the ability of electrochemical methods for analysis of trace amounts of food or other electro-active materials [21,22,23,24,25,26,27,28,29,30,31]. With the growth of new nanomaterials and their unique properties [32,33,34], the electrochemical sensors showed better ability for determination of electroactive compounds, and especially, food products [35,36,37,38,39,40]. In addition, the coupling of nanomaterials with other conductive mediators showed a powerful ability for trace level analysis of electroactive materials [41,42,43,44,45].

Vanillin is a natural phenolic product with a great smell that is extensively used in food and pharmaceutical products. This phenolic product can be synthesized by chemical methods. The high level of vanillin in food or pharmaceutical products can cause an increased risk of allergic reactions and so the control of its level is very important in food and pharmaceutical samples [46]. 

In this research, a CPE/NiO-SWCNTs and 1-butylpyridinium hexafluorophosphate modified carbon paste electrode (CPE/NiO-SWCNTs/BPrPF_6_) is employed for the electrochemical detection of vanillin in food samples. The analytical ability of CPE/NiO-SWCNTs/BPrPF6 to determine the quantity of vanillin is compared to that of recently developed technologies which use electrochemical sensors (see Table 1). In addition, the proposed sensor showed other advantages compared to previous suggested sensors such as easy preparation, low cost, and high sensitivity. 

## 2. Materials and Methods

Vanillin, mineral oil, nickel nitrate hexahydrate, graphite powder, sodium hydroxide, single wall carbon nanotubes-COOH, phosphoric acid, diethyl ether, and sulfuric acid were obtained from Sigma-Aldrich. For experimental investigation, a stock standard solution of vanillin (10 mM) was prepared daily by dissolving 0.038 g vanillin in 25 mL water solution. 

The electrochemical study was performed using the PGSTAT 302 N system. TEM (Philips CM30, 300 kV) and X-ray powder diffraction instruments were used for the investigation of NiO-SWCNTs structure and morphology. 

The NiO-SWCNTs were synthesized according to our previous recommended procedure—the chemical precipitation method with SWCNTs-COOH, nickel nitrate hexahydrate, and sodium hydroxide as precursors [52].

### 2.1. Preparation of CPE/NiO-SWCNTs/BPrPF_6_

CPE/NiO-SWCNTs/BPrPF_6_ were prepared by mixing 0.95 g of graphite powder and 0.05 g of NiO-SWCNTs in the presence of an appropriate amount of mineral oil and 1-butylpyridinium hexafluorophosphate until a uniformly wetted paste was obtained. The paste was input into the end of a glass tube in the presence of copper wire as a conductive binder.

### 2.2. Preparation of Real Sample

Coffee, milk, biscuit, and chocolate, were purchased and used for checking the ability of NiO-SWCNTs/BPrPF_6_ to perform vanillin analysis in real samples. Ten real samples were obtained from the local market and were ground using a mortar and pestle. Half a gram of powder or 0.5 mL coffee was transferred in 5 mL ethanol solution and then sonicated for 1.0 h. The obtained samples, including the vanillin extract, were centrifuged (3000× *g* rpm) for 50 min and directly used for determination of vanillin by standard addition method.

## 3. Results

### 3.1. NiO-SWCNTs Morphological and Structure Investigation

**Figure 1:**
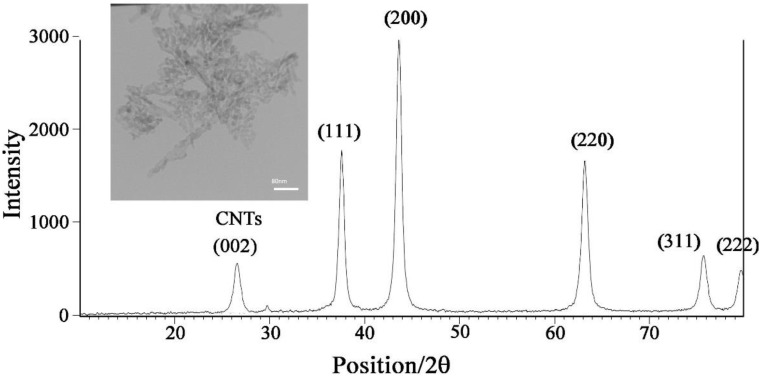
The XRD image of NiO-SWCNTs. insert TEM image of NiO-SWCNTs.

The XRD pattern of NiO-SWCNTs are presented in Figure 1 and the results confirmed the FCC structure for the NiO nanoparticle with a spherical shape and also the presence of a layer with miller index (002) at 2°~26° confirmed the presence of single wall carbon nanotubes. The TEM image of NiO-SWCNTs matches the XRD results. The NiO nanoparticle decorated the surface of single wall of carbon nanotubes (Figure 1 insert).

### 3.2. Electrochemical Behavior of Vanillin at the Surface of the Proposed Sensor

**Figure 2:**
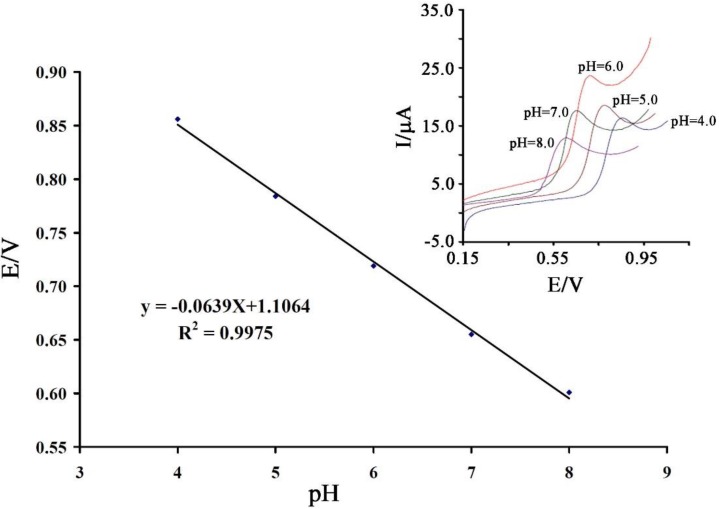
The Ep. vs. pH curve for electro-oxidation of 350 μM vanillin. Insert the linear sweep voltammograms of 700 μM vanillin at a surface of CPE/NiO-SWCNTs/BPrPF_6_ at 4.0 < pH < 8.0.

**Scheme 1:**
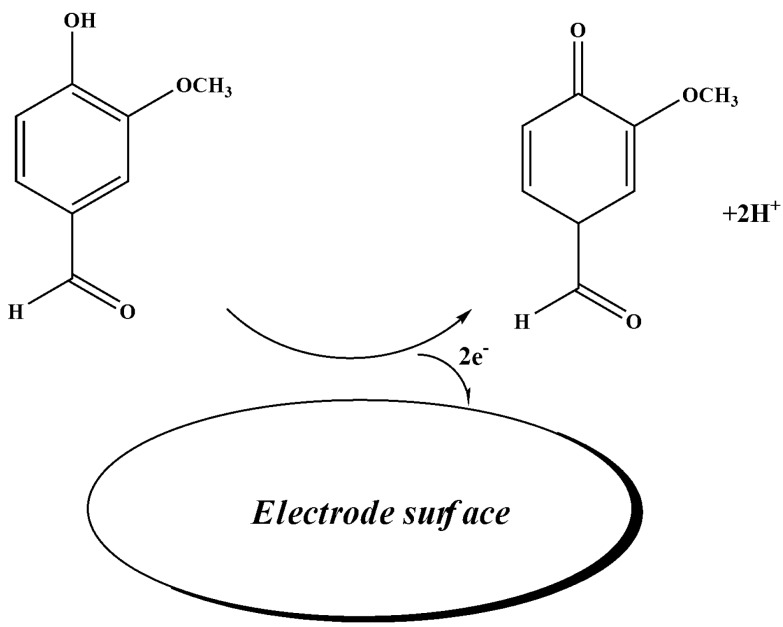
The electro-oxidation mechanism of vanillin.

The electrochemical behavior of vanillin at different pH values was investigated by the linear sweep voltammetric method (Figure 2 insert). The oxidation potential shifted to a negative value with increasing pH and the plot of E vs. pH showed a linear relation with the equation of E = −0.0639 pH + 1.1064. As can be seen, the slope of E vs. pH is near to the Nernst equation for equal value of electron and proton (see the Scheme 1).

The maximum value of current for electro-oxidation of vanillin occurred at pH = 6.0 and this condition was selected for the next steps.

**Figure 3:**
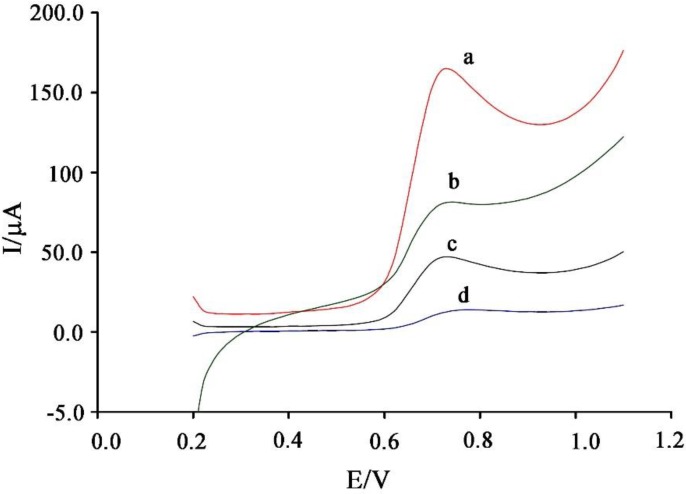
Linear sweep voltammograms of 800 μM vanillin at a surface of (**a**) CPE/NiO-SWCNTs/BPrPF_6_; (**b**) CPE/BPrPF_6_, (**c**) CPE/NiO-SWCNTs; and (**d**) CPE.

The linear sweep voltammograms of vanillin at the surface of the CPE/NiO-SWCNTs/BPrPF_6_ (curve a), CPE/BPrPF_6_ (curve b), NiO-SWCNTs (curve c), and CPE (curve d) was recorded (Figure 3). With moving of CPE to NiO-SWCNTs/BPrPF_6_, the oxidation signal of vanillin increased and the oxidation potential of vanillin decreased. This phenomenon can be attributed to the presence of NiO-SWCNTs and CPE/BPrPF_6_ at a surface of the carbon paste electrode. The NiO-SWCNTs and CPE/BPrPF_6_ improved the oxidation current of vanillin ~11.9 times and decreased the oxidation overpotential of vanillin by approximately 50 mV.

**Figure 4:**
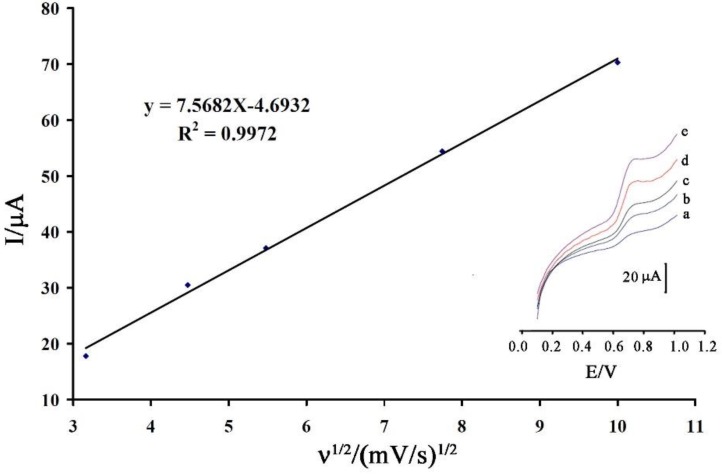
The plot of current vs. ν^1/2^ for electro-oxidation of vanillin at a surface of CPE/NiO-SWCNTs/BPrPF_6_. Insert the linear sweep voltammograms of vanillin at a surface of CPE/NiO-SWCNTs/BPrPF_6_ at scan rates of (**a**) 10.0; (**b**) 20.0; (**c**) 30.0; (**d**) 60.0; and (**e**) 100 mV/s.

The linear relation between oxidation current of vanillin and ν^1/2^ (Figure 4) confirm the diffusion process for electro-oxidation of vanillin at a surface of CPE/NiO-SWCNTs/BPrPF_6_. The oxidation potential of vanillin shifted to a positive value with increasing in-scan rates that confirm an irreversible process for electro-oxidation of vanillin (Figure 4 inert).

**Figure 5:**
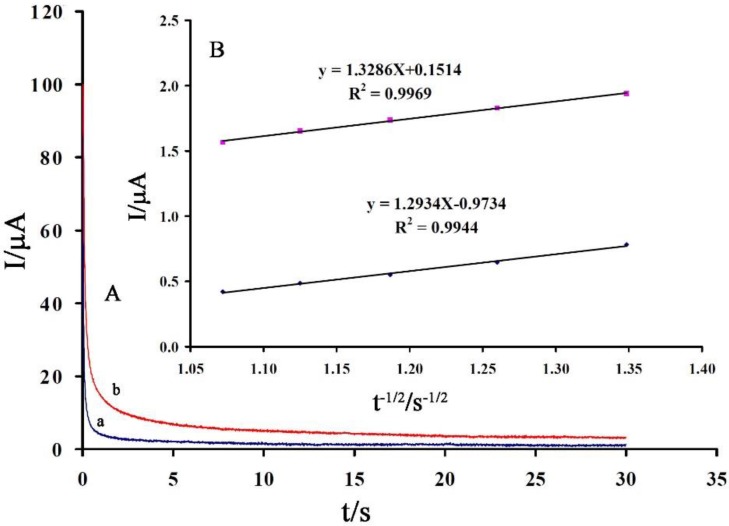
The chronoamperograms of CPE/NiO-SWCNTs/BPrPF_6_ in the presence of (**a**) 100 and (**b**) 200 μM vanillin. (**B**) Cottrell’s plot for the data from the chronoamperograms.

The value of diffusion coefficient (D) was determined by obtained data from chronoamperometric investigation (Figure 5A). 

Using the slopes from Figure 5B and Cottrell equation (Equation (1)), we determined the value of D ~ 3.57 × 10^−6^ cm^2^ s^−1^.

I = nFAD^1/2^ C π^1/2^ t^1/2^ (1)

**Figure 6:**
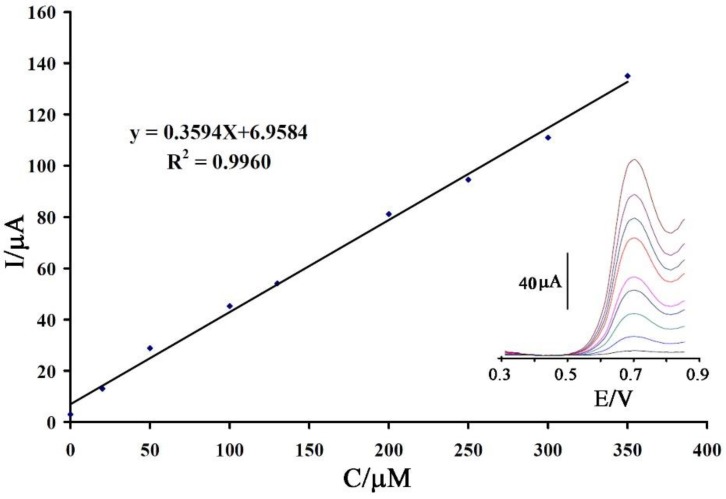
The current-concentration curve for electro-oxidation of vanillin in the range of 0.01–350.0 μM. Insert the square wave voltammograms of vanillin at surface of CPE/NiO-SWCNTs/BPrPF_6_ in the concentration range of 0.01–350.0 μM.

The square wave voltammetric method was used for investigation of the linear dynamic range and limit of detection of vanillin at a surface of CPE/NiO-SWCNTs/BPrPF_6_ (Figure 6 inset). We detected a linear dynamic range 0.01–350 μM with a detection limit of 0.007 μM (LOD = 3S_B_/m) for vanillin at a surface of CPE/NiO-SWCNTs/BPrPF_6_ (Figure 6).

The selectivity of CPE/NiO-SWCNTs/BPrPF_6_ for determination of vanillin was checked by an acceptable error of 5% in current (the obtained currents were compared before and after the addition of interference). The 1000-fold of K^+^, Na^+^, Cl^−^, glucose, and 300-fold of folic acid, vitamin B_6_, vitamin B_1_, and tartrazine had no influence on the determination of vanillin.

The ability of CPE/NiO-SWCNTs/BPrPF_6_ was checked for determination of vanillin in coffee milk, biscuit, and chocolate samples. The results are presented in Table 2. According to the results in Table 1, the CPE/NiO-SWCNTs/BPrPF_6_ was suggested as a powerful sensor for vanillin analysis in food samples.

## 4. Conclusions

This work described fabrication of a highly sensitive and new sensor for determination of vanillin in food samples. The presence of NiO-SWCNTs and BPrPF_6_ at a surface of a carbon paste electrode improved the ability of the sensor for analysis of vanillin at the nanomolar level. The NiO-SWCNTs and CPE/BPrPF_6_ improved the oxidation current of vanillin ~11.9 times and decreased the oxidation overpotential of vanillin by ~50 mV. The CPE/NiO-SWCNTs/BPrPF_6_ showed a powerful ability for determination of vanillin in food samples such as coffee milk, biscuit, and chocolate. 

## Figures and Tables

**Table 1:** The analytical data obtained by some previous voltammetric sensors for vanillin determination.

Electrode	Mediator	pH	LDR (μM)	LOD (μM)	Ref.
carbon paste	CdO/SWCNTs and ionic liquid	6.0	0.03–1200	0.009	[47]
carbon paste	CuFe_2_O_4_ nanoparticles and ionic liquid	7.0	0.1–700	0.07	[48]
glassy carbon	AuPd nanoparticles–graphene	0.1 M H_2_SO_4_	0.1–40	0.02	[49]
boron-doped diamond	anodically pre-treated	2.5	3.3–9.8	0.167	[50]
acetylene black paste	graphene–polyvinylpyrrolidone	0.1 M H_3_PO_4_	0.02–400	0.01	[51]
carbon paste	NiO-SWCNTs and ionic liquid	6.0	0.01–350	0.007	This work

**Table 2:** Determination of vanillin in real samples (*n* = 4).

Sample	Added (μM)	Expected (μM)	Founded (μM)	Recovery %
Coffee milk	---	---	4.12 ± 0.44	---
	10.00	14.12	14.43 ± 0.65	102.19
Chocolate	---	---	1.95 ± 0.24	---
	10.00	11.95	11.75 ± 0.59	98.32
Biscuit	---	---	4.56 ± 0.67	---
	10.00	14.56	14.98 ± 0.87	102.88

## References

[1] Cifuentes A. (2009). Food analysis and Foodomics. J. Chromatogr. A.

[2] Worm M., Ehlers I., Sterry W., Zuberbier T. (2000). Clinical relevance of food additives in adult patients with atopic dermatitis. Clin. Exp. Allergy.

[3] Charissou A., Ait-Ameur L., Birlouez-Aragon I. (2007). Evaluation of a gas chromatography/mass spectrometry method for the quantification of carboxymethyllysine in food samples. J. Chromatogr. A.

[4] Kenney B.F. (1991). Determination of organic acids in food samples by capillary electrophoresis. J. Chromatogr. A.

[5] Wen X., Yang Q., Yan Z., Deng Q. (2011). Determination of cadmium and copper in water and food samples by dispersive liquid–liquid microextraction combined with UV–vis spectrophotometry. Microchem. J..

[6] Yang D., Ying Y. (2011). Applications of Raman Spectroscopy in Agricultural Products and Food Analysis: A Review. Appl. Spectrosc. Rev..

[7] Shabir G.A. (2003). Validation of high-performance liquid chromatography methods for pharmaceutical analysis: Understanding the differences and similarities between validation requirements of the US Food and Drug Administration, the US Pharmacopeia and the International Conference on Harmonization. J. Chromatogr. A.

[8] Bijad M., Karimi-Maleh H., Farsi M., Shahidi S.A. (2018). An electrochemical-amplified-platform based on the nanostructure voltammetric sensor for the determination of carmoisine in the presence of tartrazine in dried fruit and soft drink samples. J. Food Meas. Charact..

[9] Sheikhshoaie M., Karimi-Maleh H., Sheikhshoaie I., Ranjbar M. (2017). Voltammetric amplified sensor employing RuO_2_ nano-road and room temperature ionic liquid for amaranth analysis in food samples. J. Mol. Liq..

[10] Baghizadeh A., Karimi-Maleh H., Khoshnama Z., Hassankhani A., Abbasghorbani M. (2015). A voltammetric sensor for simultaneous determination of vitamin C and vitamin B6 in food samples using ZrO_2_ nanoparticle/ionic liquids carbon paste electrode. Food Anal. Methods.

[11] Khaleghi F., Arab Z., Gupta V.K., Ganjali M.R., Norouzi P., Atar N., Yola M.L. (2016). Fabrication of novel electrochemical sensor for determination of vitamin C in the presence of vitamin B9 in food and pharmaceutical samples. J. Mol. Liq..

[12] Khaleghi F., Elyasi Irai A., Sadeghi R., Gupta V.K., Wen Y. (2016). A fast strategy for determination of vitamin B_9_ in food and pharmaceutical samples using an ionic liquid-modified nanostructure voltammetric sensor. Sensors.

[13] Karimi-Maleh H., Tahernejad-Javazmi F., Atar N., Yola M.L., Gupta V.K., Ensafi A.A. (2015). A novel DNA biosensor based on a pencil graphite electrode modified with polypyrrole/functionalized multiwalled carbon nanotubes for determination of 6-mercaptopurine anticancer drug. Ind. Eng. Chem. Res..

[14] Ensafi A.A., Karimi-Maleh H., Mallakpour S. (2013). A new strategy for the selective determination of glutathione in the presence of nicotinamide adenine dinucleotide (NADH) using a novel modified carbon nanotube paste electrode. Colloids Surf. B Biointerfaces.

[15] Karimi-Maleh H., Moazampour M., Ensafi A.A., Mallakpour S., Hatami M. (2014). An electrochemical nanocomposite modified carbon paste electrode as a sensor for simultaneous determination of hydrazine and phenol in water and wastewater samples. Environ. Sci. Pollut. Res. Int..

[16] Gupta V.K., Atar N., Yola M.L., Üstündağ Z., Uzun L. (2014). A novel magnetic Fe@Au core-shell nanoparticles anchored graphene oxide recyclable nanocatalyst for the reduction of nitrophenol compounds. Water Res..

[17] Sanghavi B.J., Mobin S.M., Mathur P., Lahiri G.K., Srivastava A.K. (2013). Biomimetic sensor for certain catecholamines employing copper (II) complex and silver nanoparticle modified glassy carbon paste electrode. Biosens. Bioelectron..

[18] Yola M.L., Atar N., Eren T., Karimi-Maleh H., Wang S. (2015). Sensitive and selective determination of aqueous triclosan based on gold nanoparticles on polyoxometalate/reduced graphene oxide nanohybrid. RSC Adv..

[19] Sanghavi B.J., Sitaula S., Griep M.H., Karna S.P., Ali M.F., Swami N.S. (2013). Real-time electrochemical monitoring of adenosine triphosphate in the picomolar to micromolar range using graphene-modified electrodes. Anal. Chem..

[20] Zhao Y., Gao Y., Zhan D., Liu H., Zhao Q., Kou Y., Shao Y., Li M., Zhuang Q., Zhu Z. (2005). Selective detection of dopamine in the presence of ascorbic acid and uric acid by a carbon nanotubes-ionic liquid gel modified electrode. Talanta.

[21] Beitollahi H., Raoof J.B., Karimi-Maleh H., Hosseinzadeh R. (2012). Electrochemical behavior of isoproterenol in the presence of uric acid and folic acid at a carbon paste electrode modified with 2,7-bis(ferrocenyl ethyl)fluoren-9-one and carbon nanotubes. J. Solid State Electrochem..

[22] Fouladgar M., Karimi-Maleh H. (2013). Ionic liquid/multiwall carbon nanotubes paste electrode for square wave voltammetric determination of methyldopa. Ionics.

[23] Ensafi A.A., Karimi Maleh H. (2010). A multiwall carbon nanotubes paste electrode as a sensor and ferrocenemonocarboxylic acid as a mediator for electrocatalytic determination of isoproterenol. Int. J. Electrochem. Sci..

[24] Sun W., Yang M., Jiao K. (2007). Electrocatalytic oxidation of dopamine at an ionic liquid modified carbon paste electrode and its analytical application. Anal. Bioanal. Chem..

[25] Safavi A., Maleki N., Moradlou O., Tajabadi F. (2006). Simultaneous determination of dopamine, ascorbic acid, and uric acid using carbon ionic liquid electrode. Anal. Biochem..

[26] Kuskur C.M., Swamy B.E.K., Jayadevappa H. (2018). Electrochemical behaviour of norepinephrine in the presence of paracetamol and folic acid at poly (Congo red) modified carbon paste electrode. Anal. Bioanal. Electrochem..

[27] Malhotra S., Tang Y., Varshney P.K. (2018). Non-enzymatic glucose sensor based on electrodeposition of platinum particles on polyaniline modified Pt electrode. Anal. Bioanal. Electrochem..

[28] Khalilzadeh M.A., Karimi-Maleh H., Amiri A., Gholami F. (2010). Determination of captopril in patient human urine using ferrocenemonocarboxylic acid modified carbon nanotubes paste electrode. Chin. Chem. Lett..

[29] Elyasi M., Khalilzadeh M.A., Karimi-Maleh H. (2013). High sensitive voltammetric sensor based on Pt/CNTs nanocomposite modified ionic liquid carbon paste electrode for determination of Sudan I in food samples. Food Chem..

[30] Bijad M., Karimi-Maleh H., Khalilzadeh M.A. (2013). Application of ZnO/CNTs nanocomposite ionic liquid paste electrode as a sensitive voltammetric sensor for determination of ascorbic acid in food samples. Food Anal. Methods.

[31] Najafi M., Khalilzadeh M.A., Karimi-Maleh H. (2014). A new strategy for determination of bisphenol A in the presence of Sudan I using a ZnO/CNTs/ionic liquid paste electrode in food samples. Food Chem..

[32] Sheikholeslami M. (2018). Solidification of NEPCM under the effect of magnetic field in a porous thermal energy storage enclosure using CuO nanoparticles. J. Mol. Liq..

[33] Sheikholeslami M. (2018). Numerical modeling of nano enhanced PCM solidification in an enclosure with metallic fin. J. Mol. Liq..

[34] .Sheikholeslami M. (2018). Numerical investigation for CuO-H_2_O nanofluid flow in a porous channel with magnetic field using mesoscopic method. J. Mol. Liq..

[35] Arabali V., Ebrahimi M., Abbasghorbani M., Gupta V.K., Farsi M., Ganjali M., Karimi F. (2016). Electrochemical determination of vitamin C in the presence of NADH using a CdO nanoparticle/ionic liquid modified carbon paste electrode as a sensor. J. Mol. Liq..

[36] Goyal R.N., Gupta V.K., Chatterjee S. (2008). Simultaneous determination of adenosine and inosine using single-wall carbon nanotubes modified pyrolytic graphite electrode. Talanta.

[37] Karimi-Maleh H., Tahernejad-Javazmi F., Ensafi A.A., Moradi R., Mallakpour S., Beitollahi H. (2014). A high sensitive biosensor based on fept/cnts nanocomposite/*N*-(4-hydroxyphenyl)-3,5-dinitrobenzamide modified carbon paste electrode for simultaneous determination of glutathione and piroxicam. Biosens. Bioelectron..

[38] Karimi-Maleh H., Biparva P., Hatami M. (2013). A novel modified carbon paste electrode based on nio/cnts nanocomposite and (9,10-dihydro-9,10-ethanoanthracene-11,12-dicarboximido)-4-ethylbenzene-1,2-diol as a mediator for simultaneous determination of cysteamine, nicotinamide adenine dinucleotide and folic acid. Biosens. Bioelectron..

[39] Karimi-Maleh H., Shojaei A.F., Tabatabaeian K., Karimi F., Shakeri S., Moradi R. (2016). Simultaneous determination of 6-mercaptopruine, 6-thioguanine and dasatinib as three important anticancer drugs using nanostructure voltammetric sensor employing Pt/MWCNTs and 1-butyl-3-methylimidazolium hexafluoro phosphate. Biosens. Bioelectron..

[40] Sanati A.L., Karimi-Maleh H., Abbasghorbani M. (2015). Synthesis of NiO nanoparticle and application of its in the preparation of electrochemical sensor for voltammetric determination of Nalbuphine. J. Appl. Chem..

[41] Sanati A.L., Faridbod F., Ganjali M.R. (2017). Synergic effect of graphene quantum dots and room temperature ionic liquid for the fabrication of highly sensitive voltammetric sensor for levodopa determination in the presence of serotonin. J. Mol. Liq..

[42] Sanati A.L., Faridbod F. (2017). Electrochemical Determination of Methyldopa by Graphene Quantum Dot/1-butyl-3-methylimidazolium hexafluoro phosphate Nanocomposite Electrode. Int. J. Electrochem. Sci..

[43] Ashjari M., Karimi-Maleh H., Ahmadpour F., Shabani-Nooshabadi M., Sadrnia A., Khalilzadeh M.A. (2017). Voltammetric analysis of mycophenolate mofetil in pharmaceutical samples via electrochemical nanostructure based sensor modified with ionic liquid and MgO/SWCNTs. J. Taiwan Inst. Chem. Eng..

[44] Alavi-Tabari S.A.R., Khalilzadeh M.A., Karimi-Maleh H., Zareyee D. (2018). An amplified platform nanostructure sensor for the analysis of epirubicin in the presence of topotecan as two important chemotherapy drugs for breast cancer therapy. New J. Chem..

[45] Alavi-Tabari S.A.R., Khalilzadeh M.A., Karimi-Maleh H. (2018). Simultaneous determination of doxorubicin and dasatinib as two breast anticancer drugs uses an amplified sensor with ionic liquid and ZnO nanoparticle. J. Electroanal. Chem..

[46] Van Assendelft H. (1987). Adverse drug reactions checklist. Br. Med. J..

[47] Cheraghi S., Taher M.A., Karimi-Maleh H. (2017). Highly sensitive square wave voltammetric sensor employing CdO/SWCNTs and room temperature ionic liquid for analysis of vanillin and folic acid in food samples. J. Food Compos. Anal..

[48] Khalilzadeh M.A., Arab Z. (2017). High sensitive nanostructure square wave voltammetric sensor for determination of vanillin in food samples. Curr. Anal. Chem..

[49] Jiang L., Ding Y., Jiang F., Li L., Mo F. (2014). Electrodeposited nitrogen-doped graphene/carbon nanotubes nanocomposite as enhancer for simultaneous and sensitive voltammetric determination of caffeine and vanillin. Anal. Chim. Acta.

[50] Yardım Y., Gülcan M., Şentürk Z. (2013). Determination of vanillin in commercial food product by adsorptive stripping voltammetry using a boron-doped diamond electrode. Food Chem..

[51] Deng P., Xu Z., Zeng R., Ding C. (2015). Electrochemical behavior and voltammetric determination of vanillin based on an acetylene black paste electrode modified with grapheme-polyvinylpyrrolidone composite film. Food Chem..

[52] Sanati A., Karimi-Maleh H., Badiei A., Biparva P., Ensafi A.A. (2014). A voltammetric sensor based on NiO/CNTs ionic liquid carbon paste electrode for determination of morphine in the presence of diclofenac. Mater. Sci. Eng. C.

